# Erwartungen der Generation Y an digitale Gesundheitsinnovationen

**DOI:** 10.1007/s00103-022-03567-2

**Published:** 2022-07-29

**Authors:** Thea Kreyenschulte, Sabine Bohnet-Joschko

**Affiliations:** Lehrstuhl für Management und Innovation im Gesundheitswesen, Fakultät für Wirtschaft und Gesellschaft, Universität Witten/Herdecke, Alfred-Herrhausen-Str. 50, 58448 Witten, Deutschland

**Keywords:** E-Health, Technologie, Young Professionals, Gesundheitsversorgung, Fokusgruppen, E-Health, Technology, Young Professionals, Healthcare, Focus groups

## Abstract

**Hintergrund und Ziel:**

Als „Generation Y“ werden jüngere Erwachsene bezeichnet, die im ungefähren Zeitraum von 1980–2000 geboren wurden. Der Generation wird zugeschrieben, digital affin und technisch versiert zu sein. Somit kann davon ausgegangen werden, dass bei diesen Menschen ein verstärkter Wunsch besteht, im Bedarfsfall digitale Innovationen der Gesundheitsversorgung zu nutzen. Die genauen diesbezüglichen Erwartungen sind jedoch weitestgehend unbekannt. Das Ziel dieser Arbeit ist es, die Erwartungen und Bedarfe der Generation Y genauer zu untersuchen.

**Material und Methoden:**

Zur Datenerhebung wurden im April 2021 5 Fokusgruppeninterviews mit jeweils 6 Personen zwischen 23 und 36 Jahren geführt, wobei pro Gruppe 3 Personen im Gesundheitsbereich tätig waren. Das Interviewmaterial wurde anhand der Inhaltsanalyse nach Mayring ausgewertet.

**Ergebnisse:**

Bei den Befragten bestand u. a. die Erwartung, niedrigschwellige digitale Innovationen der Gesundheitsversorgung zu nutzen. Zudem zeigte sich ein großer Bedarf an Gesundheitsinformationen. Im Vordergrund stand die Steigerung der Effizienz im Alltag, insbesondere eine Verringerung des Zeitaufwands. Einflüsse der Coronapandemie auf die Erwartungen konnten beobachtet werden.

**Diskussion:**

3 Funktionen von digitalen Anwendungen stellen sich als besonders relevant heraus: „Verwaltung“, „Tracking“ und „Information“. Zu diesen wurden Präferenzprofile der Generation Y erstellt. Passgenaue Angebote digitaler Innovationen könnten helfen, zielgruppenspezifische Gesundheitsförderung und Prävention konkreter umzusetzen und einen Mehrwert für Menschen der Generation Y zu generieren. Da der Alltag der Zielgruppe bereits von Digitalisierung und digitalen Innovationen geprägt ist, liegt hier großes Potenzial.

**Zusatzmaterial online:**

Zusätzliche Informationen sind in der Online-Version dieses Artikels (10.1007/s00103-022-03567-2) enthalten.

## Abstract (en)

**Background and aim:**

“Generation Y” is the term used to describe younger adults born between approximately 1980 and 2000. The generation is attributed with being digitally savvy and technically adept. It can thus be assumed that there is an increased desire among these people to use digital healthcare innovations when needed. However, the exact expectations in this regard are largely unknown. The aim of this study is to examine the expectations and needs of Generation Y in more detail.

**Materials and methods:**

For data collection, five focus group interviews were conducted in April 2021, each with six people between 23 and 36 years of age, with three people per group working in the health sector. The interview material was analyzed using content analysis according to Mayring.

**Results:**

One of the expectations among respondents was to use low-threshold digital healthcare innovations. There was also a great demand for health information. The focus was on increasing efficiency in everyday life, in particular a reduction in the amount of time required. Influences of the corona pandemic on expectations were observed.

**Discussion:**

Three functions of digital applications emerge as particularly relevant: “administration,” “tracking,” and “information.” For these, preference profiles of Generation Y were created. Customized solutions offered by digital innovations could help to implement target-group-specific health promotion and prevention more concretely and generate added value for Generation Y. Since the everyday life of the target group is already characterized by digitization and digital innovations, there is great potential here.

## Einleitung

Die digitale Transformation des Gesundheitswesens, und somit der Wandel durch den Einsatz neuer Technologien, bedarf umfangreicher Forschungsbemühungen, um Voraussetzungen, Zugang, Bedarfe und Wirkungen zu beschreiben [1]. Aktuell existiert vielfältige Forschung zu digitalen Anwendungen für Patient*innen in der Gesundheitsversorgung [2]. Zielgruppen sind meist chronisch Erkrankte bzw. Personen, die über längere Zeit ärztlich oder therapeutisch begleitet werden. Jüngere Menschen, die seltener von Erkrankungen betroffen sind, werden bei den aktuell untersuchten digitalen Versorgungsangeboten hingegen wenig berücksichtigt. Das Ziel der vorliegenden Untersuchung ist daher eine erste qualitative Erhebung von Bedarfen einer Zielgruppe, die im Zeitraum zwischen 1985 und 2000 geboren wurde [3–12] und einen Teil der Generation Y (ca. 1980–2000) repräsentiert.

Es handelt sich um junge, vergleichsweise gut gebildete Personen, die aktuell nur im geringen Maße Gesundheitsleistungen in Anspruch nehmen [3, 11, 13]. Die Generation Y ist nach den sogenannten „Babyboomern“ (ca. 1946–1964) derzeit die zahlenmäßig größte Generation [7, 10]. Sie wird in den nächsten Jahren einen Großteil des Arbeitsmarktes bestimmen (2025: geschätzt 75 %; [9, 10, 14–16]). Ihr wird zugeschrieben, die technologische Durchdringung in vielen Lebensbereichen zu erfahren, digital affin sowie technisch versiert zu sein [7–9, 14]. Somit besteht bei Generation Y potenziell der Bedarf, digitale Innovationen des Gesundheitswesens zu nutzen [17]. Während bereits von den Babyboomern über Generation X (ca. 1965–1980) das Gesundheitsbewusstsein stetig stieg [18], ist Generation Y ganzheitlicher ausgerichtet, kooperativ, will mit Fachkräften der Gesundheitsversorgung interagieren, auch wenn keine chronischen Erkrankungen vorliegen [3, 12, 19]. Gesundheitsdaten der Zielgruppe geben zwar Aufschluss über die Inanspruchnahme von Versorgungsleistungen – v. a. zu den Indikationen Schwangerschaft, Geburt und Wochenbett, psychische Erkrankungen sowie Verletzungen und Vergiftungen [13] –, jedoch nicht über empfundene Bedarfe und relevante Gesundheitsthemen. Deren Adressierung mittels digitaler Gesundheitsanwendungen könnte die passgenaue Versorgung im Hinblick auf eine Gesunderhaltung dieser Zielgruppe stärken [20].

Das Gesundheitsverhalten kann durch Ansprache der *Selbstwirksamkeit* eines Menschen beeinflusst werden, diese Erkenntnis ist auch für die Entwicklung digitaler Innovationen relevant [18, 21]. Das Konzept der Selbstwirksamkeit ist Teil der „sozialkognitiven Lerntheorie“ nach Bandura und bildet zusammen mit den Variablen „Wissen“, „Ziele“, „Ergebniserwartungen“, „Treiber“ und „Hindernisse“ das grundlegende Modell zur Verhaltenserklärung und -veränderung. Definiert wird Selbstwirksamkeit von Bandura als die von einer Person „empfundene Kontrolle“ über die erfolgreiche Umsetzung alltagsrelevanter Aufgaben, aber auch über ihr Gesundheitsverhalten [21, 22]. Letzteres umfasst auch die Interaktionen mit der Gesundheitsversorgung und ihren Akteuren [23]. Mit dem Wissen darüber, wie die Selbstwirksamkeit positiv beeinflusst werden kann, können zielgruppenspezifische Maßnahmen gezielt geplant und eingesetzt werden, um einen Mehrwert für die Gesundheitsversorgung zu generieren [22].

## Methodik

Zur Exploration relevanter Charakteristika von Generation Y wurden Fokusgruppendiskussionen geführt. Diese erlauben einen vergleichsweise einfachen Einblick in Zielgruppenperspektiven [24] und ermöglichen den Zugang zu Themen, über die bisher nur in geringem Maße Erkenntnisse vorliegen [25].

Die Fokusgruppendiskussionen fanden im April 2021 in 5 Gruppen à 6 Teilnehmenden, die zwischen 23 und 36 Jahre alt waren, per Videokonferenzsystem „Zoom“ statt. Die Interviews dauerten jeweils 90 min und folgten einem strukturierten Leitfaden (siehe Onlinematerial). Die Diskussionen wurden für die spätere Transkription und Analyse aufgezeichnet. Durch das Format der Videokonferenz wurde die örtliche Flexibilität aller Beteiligten ermöglicht, während der methodische Rahmen von Fokusgruppen unberührt blieb [24]. Der Fokus lag auf Alltagsthemen und relevanten Lebensbereichen, der Identifikation von Berührungspunkten mit der Gesundheitsversorgung sowie Erfahrungen mit und Erwartungen an digitale Innovationen der Gesundheitsversorgung.

### Sampling und Rekrutierung

Die Teilnehmenden wurden anhand vorab festgelegter Kriterien rekrutiert. Sie sollten berufstätig und mindestens in Besitz eines Ausbildungs- oder Bachelorabschlusses sein, andernfalls noch Studierende im Masterstudium. Jeweils 3 Teilnehmende einer Gruppe sollten aktuell im Gesundheitswesen tätig sein oder in diesem Bereich studieren, während 3 weitere einem beliebigen anderen Fachbereich angehören konnten. Diese Kombination sollte inspirieren und tiefergehende Diskussionen anregen. Informationen zu den Merkmalen der Fokusgruppenteilnehmenden finden sich in Tab. 1.

**Table 1:** 

Höchster Abschluss	Anzahl	Ø Alter	Fachbereich	Berufe und Arbeitsfelder
Master	18	29 Jahre	Gesundheitswissenschaften	Wissenschaftliche/r Mitarbeiter/in
			Gesundheitsförderung/-prävention	Psychologe/in
			Pflegewissenschaften	Angestellte/r (Konzerncontrolling; Prävention und Gesundheitsförderung)
			Sozialwissenschaften	
			Ethik und Sozialmanagement	Referent/in
			Politikwissenschaften	Projektkoordinator/in
			Psychologie	Projektplaner/in
			Gesundheitsökonomie	Führungskraft
			Volkswirtschaftslehre	Pflegemanager/in
			Grundschullehramt	Referendar/in
Bachelor	7	28 Jahre	Maschinenbau	Student/in
			Computervisualistik und Design	Webentwickler/in
			Wirtschaftsingenieurswesen	Projektleiter/in
			Mechatronik	Projektmanager/in
			Pflege	Gründer/in
			Public Health	Gesundheits- und Krankenpfleger/in
Berufsausbildung	5	25 Jahre	Medizinische/r Fachangestellte/r	Student/in
			Chirurgische/r Assistent/in	Kundenberater/in
			Gesundheits- und Krankenpfleger/in	
			IT-System-Kaufmann	

### Analyse des Interviewmaterials

Nach der Aufzeichnung der jeweiligen Fokusgruppendiskussion wurde ein wortwörtliches Transkript mithilfe der Software MAXQDA (VERBI Software, Berlin, 2021) erstellt. Es wurde eine Inhaltsanalyse, speziell die induktive Kategorienbildung, nach Mayring (2015) durchgeführt. Regel- und theoriegeleitet in ihrer Interpretation folgt die Kategorienbildung konkreten Fragestellungen, welche in weitere Unterfragestellungen gegliedert sein können. Die Fragestellungen sind präzise, theoretisch begründet und knüpfen an bereits gewonnene Forschungserkenntnisse zur Generation Y an [26]. Nachdem Textpassagen von einer Autorin codiert wurden, wurden Entscheidungen zu Codes im Team der Autorinnen diskutiert.

Sobald Gesundheit und ihre Erhaltung das Ziel sind – und nicht allein Krankheit vermieden und Mittel zu ihrer Heilung sichergestellt werden sollen –, wird auch die Bandbreite gesundheitsfördernder Mittel größer, andere Variablen müssen betrachtet und unterschiedliche Erkenntnisse einbezogen werden [22]. Daher sollte speziell das Konzept der Selbstwirksamkeit nach Bandura in der Analyse Aufschluss über Erwartungen der vorwiegend gesunden und wenig mit der klassischen Gesundheitsversorgung interagierenden Teilnehmenden an die (digitale) Gesundheitsversorgung geben.

Auf Grundlage theoriebasierter Vorannahmen wurden die in Tab. 2 aufgeführten Fragestellungen zur induktiven Kategorienbildung formuliert. Alltagsthemen wurden insbesondere deshalb thematisiert, weil Bedarfe und Berührungspunkte mit der Gesundheitsversorgung häufig nicht ohne Weiteres beobachtet oder abgefragt werden können.

**Table 2:** 

Bereich	Allgemeine Fragestellung	Spezifische Fragestellung
I.) Erwartungen an digitale Innovationen	Welche Erwartungen hat die Zielgruppe an die Gesundheitsversorgung und die digitale Gesundheitsversorgung der Zukunft?	A) In welchem Kontext kommt die Zielgruppe mit der Gesundheitsversorgung in Berührung oder würde es gerne?
		B) Welche digitalen Innovationen und darüber hinaus welche Aspekte/Eigenschaften von Innovationen sind für die Befragten relevant?
II.) Alltagsspezifische Bedarfe	Welchen Einfluss haben der Alltag der Zielgruppe sowie ihre Herausforderungen und Potenziale auf ihre Erwartungen an die digitale Gesundheitsversorgung?	C) Welche Bedarfe erlebt Generation Y in ihrem Alltag, die einen Einfluss auf die Selbstwirksamkeitserwartung haben können?

## Ergebnisse

Die Diskussionen in den Fokusgruppen gestalteten sich vielfältig. Die anhand der Inhaltsanalyse erzeugten Kategorien umfassen entsprechend den Fragestellungen folgende Gruppen: A) Gesundheitsthemen und aktuelle Berührungspunkte mit der Gesundheitsversorgung, B) digitale Innovationen der Gesundheitsversorgung und ihre (potenzielle) Nutzung sowie C) alltagsspezifische Bedarfe der Teilnehmenden mit potenziellem Einfluss auf die Selbstwirksamkeitserwartung (eine Auflistung der Kategorien findet sich in Tab. 3). Inhaltsunterstützende Zitate der Teilnehmenden wurden im Folgenden zum besseren Verständnis geglättet.

**Table 3:** 

Code-Nr.	Kategorien und Unterkategorien	Anzahl Codings
**A**	**Gesundheitsthemen bzw. aktuelle Berührungspunkte mit der Gesundheitsversorgung**	**136**
*A1*	*Gesundheitspolitik/Ausgestaltung Versorgung und Versicherung*	*17*
A1.1	Organisation und Kommunikation	13
*A2*	*Diagnostik*	*4*
*A3*	*Indikationen (allgemein)*	–
A3.1	Verletzungen	1
A3.2	Schwangerschaft	3
A3.3	Psychische Erkrankungen/mentale Gesundheit	15
A3.4	Konsum von Substanzen	3
A3.5	Bewegungsapparat	4
A3.6	Hormon‑/Sexualgesundheit	3
A3.7	Schlafgesundheit	1
A3.8	Hautgesundheit	1
A3.9	Zahngesundheit	2
*A4*	*Prävention (allgemein)*	*16*
A4.1	Zahnvorsorge	2
A4.2	Impfen	2
A4.3	Hautkrebsvorsorge	1
A4.4	Gynäkologische Vorsorge	1
A4.5	Rückengesundheit	1
*A5*	*Gesundheitsförderung (allgemein)*	*8*
A5.1	Ernährung	9
A5.2	Aktivität/Sport/Bewegung	4
A5.3	Ausgleich/Entspannung	3
*A6*	*Therapie (allgemein)*	*2*
A6.1	Physiotherapie	3
A7	Ganzheitliche Medizin/Gesundheit	7
A8	BGM (Betriebliches Gesundheitsmanagement)	10
**B**	**Digitale Innovationen der Gesundheitsversorgung und ihre (potenzielle) Nutzung**	**343**
*B1*	*Anwenderorientierung (allgemein)*	*4*
B1.1	Datenschutz/-sicherheit	16
B1.2	Praktikabilität und Service	42
B1.3	Transparenz	7
B1.4	Menschliche/persönliche Ansprechperson	11
B1.5	Preisgestaltung	5
B1.6	Funktionalität	3
B1.7	Benutzerfreundlichkeit der Anwendung	11
*B2*	*Funktionen (allgemein)*	–
B2.1	Bereitstellung von Informationen	38
B2.2	Erinnerung/Verstärkung	8
B2.3	Verwaltung	20
B2.4	Dokumentation/Tagebuchfunktionen	7
B2.5	Indikationsspezifische Funktionen	18
B2.6	Messen von Werten	16
B2.7	Assistenz und Unterstützung	10
B2.8	Vernetzung mit Leistungserbringer*innen	6
B2.9	Ergänzung klassischer Strukturen	4
*B3*	*Digitale Innovationen (allgemein)*	*4*
B3.1	Smart Devices/Wearables	10
B3.2	Apps	26
B3.3	Onlineanwendung	4
B3.4	Künstliche Intelligenz	8
B3.5	Telekonsultation	16
B3.6	Patientenakten/Aktenlösungen	9
B3.7	Implantate	6
B3.8	Robotik/Prothetik	4
B3.9	Drohnen	3
B3.10	VR/AR (Virtual/Augmented Reality)	2
B3.11	Umgebungssensorik	6
B3.12	Chatbots	1
B3.13	3‑D-Druck	3
B3.14	Genomcutting	1
*B4*	*Anbieter (allgemein)*	–
B4.1	Krankenversicherungen	7
B4.2	Geprüfter, seriöser Anbieter (nicht näher spezifiziert)	3
B4.3	Staatlich/gesetzlich	2
B4.4	Start-up/KMU (kleine und mittlere Unternehmen)	2
**C**	**Alltagsspezifische Bedarfe der Zielgruppe mit potenziellem Einfluss auf die Selbstwirksamkeitserwartung**	**176**
*C0*	*Kontrollüberzeugung (allgemein)*	*27*
*C1*	*Arbeit und Arbeitsumfeld (allgemein)*	–
C1.1	Work-Life-Balance	9
C1.2	Moderne/digitale Strukturen/Umdenken	5
C1.3	Initiative durch Arbeitgeber	2
*C2*	*Gemeinschaft/sozialer Kontakt*	*7*
*C3*	*Privat/Lifestyleaspekte (allgemein)*	–
C3.1	Effizienz/Zeitersparnis	10
C3.2	Entspannung und Ausgleich	5
C3.3	Achtsamkeit	3
C3.4	Sinnhaftigkeit	12
C3.5	Eigenverantwortung	13
C3.6	Unabhängigkeit	5
C3.7	Nachhaltigkeit	6
C3.8	Bewegung/Sport	1
C3.9	Organisation freier Zeit/Zeitmanagement	1
*C4*	*Zwischenmenschliche Faktoren (allgemein)*	–
C4.1	Kommunikation auf Augenhöhe	5
C4.2	Mehr Zeit und Feedback	11
C4.3	Wertschätzung	5
*C5*	*Information*	*20*
*C6*	*Chancengleichheit*	*4*
*C7*	*Sicherheit und Vertrauen*	*12*
*C8*	*Transparenz*	*13*
*C9*	*Positive Einstellung*	*1*

### A) Gesundheitsthemen und aktuelle Berührungspunkte mit der Gesundheitsversorgung

Die in *Gruppe A)* formulierten Kategorien betreffen Bereiche des Versorgungsprozesses, spezifische Indikationen und die Ausgestaltung der Gesundheitsversorgung. Den Teilnehmenden waren insbesondere die Themen *Gesundheitspolitik und Ausgestaltung von Versorgung und Versicherung (A1), Organisation und Kommunikation von Gesundheitsthemen (A1.1), Prävention (A4), Betriebliches Gesundheitsmanagement (A8) sowie psychische Erkrankungen bzw. mentale Gesundheit (A3.3)* wichtig.

Die gesundheitspolitischen Themen betrafen insbesondere das duale Krankenversicherungssystem von gesetzlicher und privater Krankenversicherung und seine Auswirkungen auf die Gesellschaft. Es fielen Begriffe wie „Zwei-Klassen-Gesellschaft“ oder „mangelnde Solidarität“. Bezogen auf die Interaktion zwischen Fachkraft und Patient*in bestand der Wunsch nach einer Reduktion hierarchischer, asymmetrischer Kommunikation, um wahrgenommene Ineffizienzen im Verständnis zu beseitigen. Es existierte die Sorge, dass der aktuelle individuelle Informationsstand nicht ausreichen könnte, um auf Augenhöhe mit Fachkräften zu kommunizieren, Nutzen und Risiken abzuschätzen oder relevante Versorgungsangebote zu erkennen.

„Man weiß ganz schwer, was wirklich richtig für einen ist. Die im Krankenhaus sagen, du musst einmal die Woche zur Nachsorge, dann sagen die ambulant, du sollst alle zwei Wochen kommen. Deswegen ist mir das jetzt so das erste Mal aufgefallen, dass man da sehr abhängig auf eine Art ist.“

Ähnlich war es im Präventionsbereich. Die Teilnehmenden wünschten sich in der Versorgung eine Gesundheitsorientierung, inklusive Beratung zu Präventionsthemen und -maßnahmen. Auch wurde im Arbeitsumfeld ein stärkerer Fokus auf Gesundheitsthemen erwartet. Derzeit herrsche dort ein Mangel an Angeboten sowie das Gefühl, dass Arbeitgeber dem Thema Gesundheit am Arbeitsplatz zu wenig Relevanz beimessen. Dies münde in einem Gefühl von Unverständnis und mangelnder Wertschätzung.

„Da sehe ich aber tatsächlich auch den Arbeitgeber mehr in der Verpflichtung, hier Angebote zu unterbreiten für Mitarbeiter*innen, in Richtung betriebliche Gesundheitsförderung. Also es muss deutlich mehr sein, als ein Obstkorb, wenn gerade die Grippesaison wieder anfängt.“

Eine spezifische Indikation, die für die Teilnehmenden relevant war, waren psychische Erkrankungen. Die Befragten berichteten, dass in ihrem Umfeld psychische Leiden aufträten, die es zu enttabuisieren gelte. Insbesondere Stress, Depression oder Burn-out wurden thematisiert ebenso wie der Wunsch mental gesund zu bleiben. Weitere Indikationen, bestehende spezifische Erkrankungen oder gar chronische Leiden wurden nicht diskutiert.

### B) Digitale Innovationen der Gesundheitsversorgung und ihre (potenzielle) Nutzung

*Gruppe B)* umfasst Kategorien zu digitalen Innovationen. Hier war von Interesse, welche digitalen Anwendungen der Gesundheitsversorgung bereits genutzt werden und welche Anforderungen an diese bestehen. Am häufigsten wurden *Apps (B3.2), Telekonsultation (B3.5), Smart Devices (B3.1) *und* Patientenakten (B3.6)* als Schnittstellen digitaler Gesundheitsversorgung genannt.

Unter Smart Devices fassten die Teilnehmenden insbesondere Smartwatches. Vorteile der Nutzung wurden darin gesehen, perspektivisch Diagnosen aus der Ferne gestellt sowie aufgrund gesammelter Daten ein stärkeres Sicherheitsgefühl zu bekommen. Neben schnell zugänglichen Apps schätzten die Befragten auch die zeitliche und räumliche Flexibilität, die durch Telekonsultationen ermöglicht wird. Es bestand keine Bereitschaft, lange auf einen Termin zu warten oder Wege auf sich zu nehmen. Laut den Teilnehmenden wäre es bei manchen Erkrankungen auch gar nicht notwendig, eine ärztliche Praxis aufzusuchen.

Die Priorisierung von Work-Life-Balance sowie von Effizienz und Produktivität spiegelte sich auch in der Einstellung der Befragten zu elektronischen Patientenakten wider. Aktenlösungen würden u. a. dazu dienen, einen Überblick über Behandlungen zu gewinnen, die Anamnese zu erleichtern und Zeit zu sparen.

„Das Thema eAkte finde ich tatsächlich ganz spannend. Weil ich nicht mehr nachvollziehen kann, wann ich bei welchem Arzt war, wenn ich Gesundheitsbögen ausfüllen muss. Wann war mein Kreuzbandriss? Weiß ich nicht mehr, vor zehn oder zwölf Jahren, irgendwie so etwas. Aber ich kann mir das nicht alles merken.“

Neben Schnittstellen digitaler Versorgung wurden Anwendungskriterien diskutiert. Für die Befragten war bezüglich der Anwenderorientierung digitaler Innovationen das Kriterium *B1.2 Praktikabilität und Service *besonders relevant. Praktikabilität bedeute, dass alles mit „einem Klick“ automatisiert abläuft. Belange sollten tagesaktuell adressierbar und Hinweise zu verwandten Themen oder Anliegen erfolgen. Ebenfalls häufig wurde* Datenschutz/-sicherheit (B1.1) *als Präferenz der Teilnehmenden genannt. Es wurde erklärt, dass Gesundheitsdaten die einzigen Informationen wären, die großen Tech-Firmen noch nicht vollständig vorlägen, und daher deren Interesse daran wahrscheinlich groß sei. Die Befragten beklagten, dass einmal übertragene Daten nicht mehr einsehbar wären und ihre Verwendung nicht nachvollziehbar wäre. Bezüglich der Anwenderorientierung bestand bei den Teilnehmenden zudem der Wunsch nach menschlichen bzw. persönlichen Ansprechpartner*innen (B1.4) – spätestens in letzter Instanz. Sobald es um Krankheit und nicht um Gesundheitsförderung gehe, würden menschliche Ansprechpartner*innen relevant.

„Im Zweifel sollte es immer einen persönlichen Ansprechpartner geben. Man weiß nie, wie groß die Krise der Person gerade ist. Also irgendwie muss da schon etwas im Hintergrund sein.“

Mit Abstand die relevanteste Funktion digitaler Anwendungen war für die Befragten das *Bereitstellen von Informationen (B2.1).* In der Weitergabe von Informationen über den Gesundheitszustand bestehe das Potenzial, frühzeitig präventiv tätig zu werden. Information diene dem Empowerment, dem Sicherheitsgefühl und der Vertrauensstärkung, aber auch dazu, mehr über sich selbst und den Körper zu erfahren oder eine Behandlung sinnvoll zu ergänzen.

„Also, wenn dann jetzt ein akuter Fall wäre, würde ich am liebsten eine App nutzen, wo man schon so etwas wie einen Kompass hat. Also Frage und Antwort, wo ich schon in die richtige Richtung geleitet werde und wo auch direkt ein Termin, am besten automatisiert, beim Arzt gemacht werden kann.“

Darüber hinaus interessierten sich die Befragten für die Funktionen *B2.3 Verwaltung, B2.5 indikationsspezifische Funktionen* und *B2.6 Messen von Werten*. Die Möglichkeit der digitalen Verwaltung war in jedem Fall relevant, egal ob bei Gesundheit oder Krankheit. Die Teilnehmenden gaben an, sich nicht länger als nötig mit verwaltenden Tätigkeiten beschäftigen zu wollen und die Tatsache zu schätzen, dass es bereits datensichere Angebote der Krankenversicherungen gibt. Die Krankenversicherungen sind laut den Befragten auch am ehesten potenzielle Anbieter digitaler Innovationen *(B4.1*). Die Teilnehmenden äußerten Interesse, anhand digitaler Aufzeichnungen stärker mit Krankenkassen in Interaktion zu gehen und individuelle Angebote anhand des (gesunden) Verhaltens herauszuarbeiten.

### C) Alltagsspezifische Bedarfe der Teilnehmenden mit potenziellem Einfluss auf die Selbstwirksamkeitserwartung

Kategorien der *Gruppe C)* bilden Bedarfe der Teilnehmenden ab, die in ihrem Alltag entstehen und einen Einfluss auf die wahrgenommene Selbstwirksamkeit haben können. Selbstwirksamkeit im Zusammenhang mit Gesundheit wird z. B. geprägt durch individuelle Fähigkeiten oder Wissen, welche zum Zweck eines gesundheitsbewussten Verhaltens eingesetzt werden können [21].

Bei den Diskussionen hat sich gezeigt, dass es den Befragten vor allem in Hinblick auf (Behandlungs‑)Fehler an *Kontrollüberzeugung* (*C0*) mangelt. Aufgrund fehlender Information und eingeschränkter Einflussmöglichkeiten könnten z. B. mögliche Komplikationen vorher nicht gut abgeschätzt werden. Auch in Hinblick auf digitale Anwendungen und die damit verbundene Preisgabe von Daten werde die Kontrollüberzeugung eingeschränkt.

„Ich denke, das kann insbesondere auch ein Problem unserer Generation und unseres Alters sein. Dass wir verhältnismäßig wenig chronische Krankheiten haben und man dann einfach nicht besonders ernst genommen wird. Man geht zum Arzt und sagt „Ich hätte jetzt gerne mal ein großes Blutbild, weil irgendwie geht es mir nicht gut und ich will, dass jetzt jemand nach Ursachen forscht“. Gefühlt muss es dann schon bis zum Äußersten kommen.“

In weiteren Kategorien zur Fragestellung C) konnten private bzw. Lifestyleaspekte der Teilnehmenden identifiziert werden. Es fielen besonders die Bedarfe an* C3.4 Sinnhaftigkeit, C3.5 Eigenverantwortung sowie C3.1 Effizienz/Zeitersparnis* ins Gewicht. Die Befragten empfanden die aktuelle Situation als ineffizient, weshalb organisatorische Tätigkeiten besser gebündelt digital stattfinden sollten.

„Dass ich nicht zum Postkasten gehen muss, um meiner Krankenkasse den gelben Schein von meinem Arzt zu schicken. Also da finde ich, ist ein riesen Erleichterungspotenzial, was völlig unabhängig davon ist, wie gesund oder wie krank, oder was für ein Anwendertyp ich überhaupt bin.“

Darüber hinaus äußerten die Befragten Zweifel an Datenschutzpraktiken, die vor Ort in Praxen und Kliniken herrschen. Namen oder Symptome würden beispielsweise für Fremde hörbar kommuniziert. Im Gegensatz dazu würden für digitale Anwendungen strenge Datenschutzargumente diskutiert, deren Sinnhaftigkeit die Befragten bezweifelten.

Die Teilnehmenden wünschten sich mehr Eigenverantwortung und übernahmen diese bereits an vielen Stellen, z. B. in Form von Recherche oder Tracking. Entsprechend dem Wunsch nach Eigenverantwortung wurde erwartet, dass möglichst viele *Informationen* (C5) zu gesundheitsrelevanten Themen, Angeboten oder zur Organisation zusätzlich zur Eigenrecherche von Behandlern und Versicherungen bereitgestellt werden.

In Hinblick auf zwischenmenschliche Faktoren wünschten sich die Befragten vor allem *mehr Zeit und Feedback (C4.2)* in der Interaktion mit medizinischem Fachpersonal. Es wurde bemängelt, dass es an Zeit fehle, sich mit Versorger*innen auszutauschen, um ein adäquates Feedback zu erhalten und sich ernst genommen zu fühlen. An dieser Stelle nimmt die Selbstwirksamkeitserwartung eher ab.

In Bezug auf das Arbeitsumfeld berichteten die Teilnehmenden am häufigsten über Bedarfe hinsichtlich einer *Work-Life-Balance (C1.1)*. Sie betonten die Gesundheitsaspekte von Freizeit und merkten an, dass eine gute Gesundheit nicht allein der Erwerbsarbeit dienen sollte.

„Also ich glaube, großes Thema bei uns ist Work-Life-Balance. Und das bedeutet eben nicht, dass die Gesundheit dafür da ist, mehr bei der Arbeit zu leisten, sondern auch, mehr von seiner freien Zeit und generell auch ein besseres Leben zu haben.“

Abschließend wurden auch die Kategorien *C7 Sicherheit und Vertrauen *sowie* C8 Transparenz* im Alltag als relevant erachtet. Hier waren insbesondere Sicherheit in Form von Datensicherheit und das Wissen um die Verwendung digital erhobener Daten gemeint, die die Selbstwirksamkeitserwartung steigern können.

### Digitale Gesundheitsversorgung in der Coronapandemie

Obwohl die Erwartungen der Teilnehmenden an eine digitale Gesundheitsversorgung unabhängig von der Coronapandemie betrachtet wurden, kam das Thema in den Fokusgruppen zur Sprache. Die Teilnehmenden beschrieben den Einfluss der Pandemie auf ihren Alltag und im Weiteren auch auf die Nutzung digitaler Anwendungen.

Demnach hat sich während der Pandemie gezeigt, dass Praxisbesuche bei niedrigschwelligen Anliegen nicht immer notwendig sind und dass digitale Anwendungen, wie z. B. Telemedizin, gute Alternativen darstellen. Sie werden inzwischen als sinnvoll angesehen und wertgeschätzt, auch über die Pandemie und ihre Einschränkungen hinaus. Es herrsche mehr „digitale Normalität“. Zudem gaben die Befragten an, dass digitale Innovationen zum Infektionsschutz beitragen können, wenn Praxisbesuche mit erhöhter Ansteckungsgefahr wegfallen. Gleichzeitig werden digitale Lösungen auch als alltagsentlastend eingestuft.

Die Teilnehmenden nahmen während der Pandemie ein gesteigertes Angebot digitaler Lösungen wahr und gingen davon aus, dass die Pandemie auch weiterhin ein Treiber für digitale Anwendungen des Gesundheitswesens und insbesondere der Telemedizin sein wird.

Auf der anderen Seite zeigte sich jedoch auch eine Verdrossenheit, was die digitale Interaktion betrifft. Die Notwendigkeit digitaler Kommunikation aufgrund der Pandemie wurde im Alltag häufig als belastend empfunden. Es wurden diesbezügliche Gefahren für die mentale Gesundheit diskutiert und die Pandemie als Faktor für die Steigerung von Leistungsdruck gesehen.

## Diskussion

Individuelle Werte und Einstellungen beeinflussen Bedarfe und die Inanspruchnahme von Angeboten der Gesundheitsversorgung. Zur Erhebung von Motiven und Erwartungen sind qualitative Forschungsansätze gut geeignet [27]. Für die Teilnehmenden unserer Untersuchung konnten wir Bedarfe identifizieren, die teilweise über bestehende Angebote hinausgehen. Gleichzeitig spiegeln sie die in anderen Studien beschriebenen Charakteristika der Generation Y wider [3, 5, 8, 11]. So wie im Kontext des Erwerbslebens Sinnhaftigkeit, Eigenverantwortung und Flexibilität angestrebt werden, äußerten die Fokusgruppenteilnehmenden diese Bedarfe auch bezogen auf Gesundheit und Versorgung. Digitale Innovationen können potenziell dabei unterstützen, mehr Kontrolle über alltägliche oder versorgungsrelevante Gewohnheiten oder Herausforderungen zu erlangen, effizienter zu handeln und somit die Selbstwirksamkeitserwartung zu steigern. Da ein großer Teil des Alltags der Generation Y bereits von Digitalisierung geprägt wird, liegt hier großes Potenzial für die Anwendung digitaler Lösungen.

Der generationsspezifische Ansatz bietet sich zur Einordnung der Ergebnisse besonders an [19, 28, 29]. Personen einer Generation weisen ähnliche Charakteristika, Werte, Überzeugungen sowie soziale, politische und technologische Erfahrungen auf, die das Verhalten beeinflussen [19, 28]. Untersuchungen zeigen, dass diese Unterschiede in verschiedensten Lebensbereichen existieren, so auch im Gesundheitsbewusstsein und -verhalten. Die Generation der Babyboomer (ca. 1946–1964) ist durch hohe Arbeitsmoral und Resilienz geprägt. Sie kam erst später im Leben mit Digitalisierung in Berührung. Heute stellt sie das Gesundheitssystem mit eintretenden chronischen Erkrankungen und gesundheitlichen Beschwerden vor Herausforderungen [18]. Generation X (ca. 1965–1980) wird großer Pragmatismus zugeschrieben. Ihre Digitalkompetenz bezieht sich vor allem auf den Umgang mit PCs und die Smartphonekommunikation. Bezogen auf ihre Gesundheit weist sie ein hohes Vertrauen in medizinisches Fachpersonal auf [28]. Generation Y (ca. 1980–2000), die Gegenstand unserer Untersuchung war, weist bereits eine hohe Technikkompetenz auf, ist gut vernetzt und möchte aktiv mitgestalten [6, 30]. Feedback von Gesundheitsfachkräften reicht ihr oft nicht. Die nachfolgende Generation Z (ca. 2000–2012) ist noch sehr jung und äußert noch weniger Gesundheitsbedarfe als Generation Y. Sie kennt eine Welt ohne Internet, Onlinekommunikation und mobile Verfügbarkeit nicht [10, 31].

Es liegt nahe, dass sich digitale Gesundheitsversorgung zunächst auf Indikationen oder die Unterstützung chronisch erkrankter Zielgruppen konzentriert. Ein aktuelles Scoping-Review zeigt, dass international bereits eine Vielfalt digitaler Gesundheitsanwendungen zur Nutzung durch Patient*innen existiert. Dabei wird der Therapiebereich fokussiert durch Angebote zu Bildung, Selbstmanagement, Symptommanagement und Behandlung [2]. Die Generationen Babyboomer und X werden ihre Bedarfe hier vermutlich gut abgebildet sehen. Hingegen sind für Generation Y digitale Gesundheitsanwendungen von Interesse, die Prävention und Gesundheitsförderung niedrigschwellig unterstützen und Organisation erleichtern. Der Vergleich mit der jüngeren Zielgruppe der Generation Z zeigt einen Fokus auf Gesundheits‑, Fitness- und Lifestyleaspekte [32].

Auf konkrete digitale Anwendungen bezogen ist erkennbar, dass diese zielgruppenspezifisch unterschiedlichen Intentionen folgen. Elektronische Patientenakten sind laut Fokusgruppenanalyse auch für Gesunde interessant, werden bisher jedoch insbesondere mit einem Nutzen für erkrankte Patient*innen verbunden [33]. Insbesondere jüngere Generationen handeln in der Gesundheitsversorgung eher wie Verbraucher*innen, die auf Basis einer Interaktion mit Versorger*innen oder Versicherungen z. B. weitere Ratschläge oder Empfehlungen erhalten möchten. Daher könnten künftig Prinzipien der Plattformökonomie (auf die Nutzer*innen ausgerichtete, ganzheitliche Onlinegeschäftsmodelle) zum Tragen kommen [34]. Apps und Smart Devices sind bei aktiven, gesunden Personengruppen ebenso beliebt wie bei Menschen, die Informationen über ihre Gesundheit erhalten wollen [32]. Telemedizin ist ebenfalls generationsübergreifend attraktiv, um Hürden im Kontakt mit der Gesundheitsversorgung zu reduzieren. Der Unterschied liegt bei Generation Y meist in der Intention, Zeit und Aufwand einzusparen [34]. Sie ist mit einer Vereinfachung durch Technologie aufgewachsen [19] und erwartet dies auch im Gesundheitswesen [34]. Für ältere oder erkrankte Zielgruppen könnten indikationsspezifische Innovationen an Bedeutung gewinnen, da sie eine Interaktion mit medizinischem Fachpersonal ermöglichen. Beispiele können Virtual Reality in der Rehabilitation [35] oder Chatbots als Mittel zur Adhärenz bei chronischer Erkrankung [36, 37] sein.

### Präferenzprofile der Generation Y

**Figure 1:**
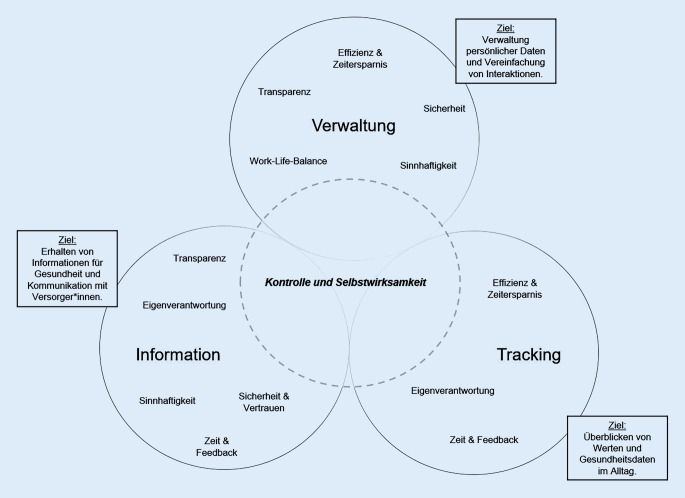

Aus dem analysierten Interviewmaterial können 3 Präferenzprofile der Generation Y abgeleitet werden. Diese beziehen sich auf die von den Fokusgruppenteilnehmenden am häufigsten genannten Funktionen von digitalen Anwendungen: Verwaltung, Tracking und Information (Abb. 1). In den Profilen sind die entsprechenden Bedarfe und Ziele der Generation Y enthalten. Die Profile weisen als Gemeinsamkeit den Bedarf an Kontrolle und Selbstwirksamkeit durch digitale Innovationen auf.

Das Präferenzprofil *Verwaltung* umfasst die Anwendung digitaler Innovationen wie Telekonsultationen oder Patientenakten zur Speicherung, Übersicht und Handhabung persönlicher Versorgungsdaten. Eine entsprechende digitale Innovation, welche bereits viele Funktionen bietet und gesetzmäßig angeboten wird, ist die elektronische Patientenakte [38].

Im Bereich *Tracking* sahen die Fokusgruppenteilnehmenden insbesondere Potenzial in Wearables und Apps, die ihnen präventive oder Lifestylehinweise (z. B. Bewegung, Zyklus) geben. Über ein vom Bundesinstitut für Arzneimittel und Medizinprodukte (BfArM) geführtes Register zur Prüfung der Wirksamkeit und Aufnahme in die Regelversorgung erfolgt seit 2020 das Angebot digitaler Gesundheitsanwendungen (DiGAs). Aktuell sind hier insgesamt 31 DiGAs gelistet, davon allein 14 im Bereich psychischer Gesundheit [39].

In Bezug auf das Präferenzprofil *Information* beschäftigte die Teilnehmenden, wo sie Gesundheitsinformationen zu relevanten Themen erhalten. Zur Einholung gesicherten Gesundheitswissens stellt der Bund eine Website bereit, welche gebündelt und transparent Informationen bietet [40].

Aus den 3 Profilen können zielgruppenspezifische Eigenschaften digitaler Innovationen abgeleitet werden, die zur Gesundheitsförderung und Unabhängigkeit vom klassischen Gesundheitssystem beitragen. Niedrigschwellige digitale Anwendungen können das Gesundheitsverhalten der Generation Y auch durch die Ansprache der Selbstwirksamkeit beeinflussen [18] und sie damit zu zielgruppenspezifischen, insbesondere präventiven und gesundheitsfördernden Maßnahmen anleiten oder sie bei deren Organisation unterstützen.

Während diese abgestimmten Maßnahmen, ebenso wie Organisation und Verwaltung, auf digitalem Weg stattfinden und somit den Alltag vereinfachen können, sollten einige der wenigen Berührungspunkte mit der Gesundheitsversorgung weiterhin klassisch erfolgen. Durch die Coronapandemie wurde deutlich, dass auch bei digital affinen Zielgruppen ein Übergang hin zur Verdrossenheit bei der digitalen Interaktion zusammen mit dem Bedarf an menschlichen Ansprechpersonen existiert.

### Limitationen

Wie häufig in qualitativen Untersuchungen sind die Fokusgruppen mit ihrer Teilnehmendenzahl von insgesamt 30 Personen nicht repräsentativ. Gesundheitsthemen der Generation Y sind, insbesondere aufgrund des gewählten Samplings, eventuell nicht in ihrer tatsächlichen Vielfalt vertreten. Befragt wurden in dieser Untersuchung ausschließlich Personen mit einem Berufs- oder Universitätsabschluss, weshalb Ergebnisse nicht auf alle Bevölkerungsschichten der Generation Y übertragbar sind. Auch fand aufgrund des übersichtlichen Datensatzes keine weitere unabhängige Codierung statt. Obwohl insgesamt sehr diverse Diskussionen geführt wurden, sind diese nur ein Ausschnitt aus möglichen Ansichten und Meinungen. Somit bleibt ein Vergleich mit anderen Zielgruppen schwierig. Auch kann nicht von einem einheitlichen Verständnis digitaler Innovationen ausgegangen werden – wie diese im Einzelnen von der Zielgruppe definiert werden, ist nicht abschließend geklärt.

### Schlussfolgerungen

Mit den Ergebnissen dieser Untersuchung kann ein Schritt hin zu einer adäquateren Berücksichtigung der Generation Y als Zielgruppe digitaler Gesundheitsversorgung erfolgen. Demnach sollten beispielsweise Gesundheitsthemen wie mentale Gesundheit, Prävention oder betriebliches Gesundheitsmanagement stärker in digitalen Anwendungen berücksichtigt werden. Wie dargestellt, verweisen insbesondere Alltagsbelange, entsprechende Herausforderungen und empfundene Potenziale auf Bedarfe der Zielgruppe. Diese umfassen u. a. die Bereitstellung und den Zugang zu einer Vielzahl an Informationen, Möglichkeiten in Hinblick auf Effizienzsteigerung und Work-Life-Balance sowie Eigenverantwortung und gleichberechtigte Kommunikation zur Steigerung der wahrgenommenen Kontrolle und Selbstwirksamkeit.

Die Ergebnisse sind sowohl für Forschung als auch Praxis relevant. So erhalten z. B. Krankenversicherungen Anhaltspunkte für Präferenzen und Bedarfe der Zielgruppe, die durch digitale Innovationen angesprochen werden könnten. Auch für ein weiteres großes Thema der Generation, die Erwerbsarbeit, lassen sich Implikationen ableiten. Aktive Gesundheitsvorsorge wird gerade in Industrien wichtiger, in denen Generation Y die Schlüsselstellen der Unternehmen besetzt. Der Arbeitsplatz ist ein zentraler Ort, um die Gesundheit der Mitarbeitenden durch das flexible Angebot digitaler Anwendungen zu adressieren. Die Ergebnisse der Fokusgruppendiskussionen können in einem nächsten Schritt zur Hypothesenbildung genutzt werden, um die Bedarfe und somit Erwartungen der Zielgruppe noch tiefergehender zu ergründen.

## Supplementary Information




