# Supplementary material for: Expectations of generation Y for digital health innovations
Source: Bundesgesundheitsblatt Gesundheitsforschung Gesundheitsschutz. 2022 Jul 29;65(9):881–90. [Article in German] doi: 10.1007/s00103-022-03567-2 (PMC9436845; doi:10.1007/s00103-022-03567-2)
Supplement: Supplementary file 1 [file 103_2022_3567_MOESM1_ESM.pdf]

Bundesgesundheitsblatt – Gesundheitsforschung – Gesundheitsschutz, Ausgabe 9/2022

Onlinematerial zum Beitrag:

Erwartungen der Generation Y an digitale Gesundheitsinnovationen

Thea Kreyenschulte[1], Sabine Bohnet-Joschko[1]

[1] Universität Witten/Herdecke, Fakultät für Wirtschaft und Gesellschaft, Lehrstuhl für Management und Innovation im Gesundheitswesen, Witten, Deutschland

Korrespondenzadresse

Thea Kreyenschulte
Lehrstuhl für Management und Innovation im Gesundheitswesen
Fakultät für Wirtschaft und Gesellschaft
Universität Witten/Herdecke
Alfred-Herrhausen-Straße 50
58448 Witten
thea.kreyenschulte@uni-wh.de

Onlinematerial: Interviewleitfaden für die Fokusgruppendiskussionen

**Ablauf, Thema**	**Fragen**
Vorstellung	Name, Alter, Schlagwort zum Thema?
Relevanz einzelner Lebensbereiche	Blick auf den Alltag: Was ist bzw. welche Lebensbereiche sind aktuell besonders relevant für euch?<br><br>*Optionale Anschlussfrage: Was ist vielleicht herausfordernd oder wo wollt ihr wiederum volles Potenzial ausschöpfen?*
Gesundheitsaspekt	Welche Rolle spielt das Thema Gesundheit im Alltag für euch?<br><br>*Optionale Anschlussfrage: Was für Gesundheitsthemen sind für euch bzw. eure Generation relevant?*<br>*Optionale Anschlussfrage: Worauf hat Gesundheit eurer Meinung nach Einfluss in eurem Leben?*
**INPUT I**<br>Möglichkeiten digitaler Gesundheitsversorgung als Inspiration und Diskussionsgrundlage<br><br>(PowerPoint-Präsentation)	Was davon kennt ihr? Wozu würdet ihr digitale Gesundheitsanwendungen wie diese nutzen?
Anschlussfrage	Inwiefern glaubt ihr, könnten diese digitalen Anwendungen euch dabei unterstützen, eure aktuellen Ziele und Herausforderungen zu meistern?
Spezifikation digitaler Gesundheitsangebote durch Zielgruppe<br>(Breakout-Rooms in Zoom: 3 Minuten)	Ihr benötigt medizinische oder therapeutische Hilfe/ ihr seid erkrankt: welche Art digitaler Gesundheitsangebote nutzt ihr und warum?
**INPUT II**<br>Kurzer Input zu potenziellen Zukunftsszenarien<br><br>(PowerPoint-Präsentation)	Ihr schaut in das Jahr 2035: Was wäre ein Anwendungsszenario, in dem ihr Gebrauch von digitalen Angeboten machen würdet?
Präferenzen und Einflussfaktoren	Was wäre euch zusammenfassend allgemein wichtig bei digitalen Angeboten (Produkten, Prozessen, Services)?<br><br>*Optionale Anschlussfrage: Wer könnte euch solche anbieten?*
